# Impact of feeding volumes in the first 24 h of life on neonatal feeding intolerance

**DOI:** 10.3389/fped.2023.1245947

**Published:** 2023-08-29

**Authors:** Navin Kumar, Igbagbosanmi Oredein, Mohammed Al-Nahar, Nathalee Harris, Venkatesh Sampath

**Affiliations:** ^1^ Division of Neonatology, Hurley Children’s Hospital, Flint, MI, United States; ^2^ Division of Neonatology, Children’s Mercy Hospital, Kansas City, MO, United States

**Keywords:** formula supplementation, feeding variability, feeding intolerance, formula switch, initial volume of feeding

## Abstract

**Objective:**

This study investigates whether volumes of intake in the first 24 h of life (24 HOL), in relation to birth weight (BW) and gestational age (GA), impact neonatal feeding intolerance (FI).

**Methods:**

This study employed a retrospective chart review of 6,650 infants born at ≥35 weeks. The volumes of each formula feed per kg BW in the first 24 HOL were assessed. FI was defined as evidenced by chart documentation of emesis, abdominal distension, abdominal x-ray, and/or switching to a sensitive formula.

**Results:**

Overall, the maximum volume of formula intake per feed was inversely correlated with GA and was higher in infants with FI (*β* = −1.39, *p* < 0.001) compared with infants without FI (*β* = −1.28, *p* < 0.001). The odds of emesis in late preterm infants with first feeding of >8 ml/kg [adjusted odds ratio (AOR) = 2.5, 95% confidence interval (CI): 1.4–4.6] and formula switching in the exclusively formula-fed group with volumes >10.5 ml/kg [AOR =  2.2, 95% CI (1.8–2.6)] were high. In the breastfeeding group, the odds of FI increased by 2.8-, 4.6-, and 5.2-fold with 5–10, 10–15, and >15 ml/kg of supplementations, respectively.

**Conclusion:**

A higher volume of intake in relation to BW often exceeds the physiological stomach capacity of newborns and is associated with early FI. Optimizing early feeding volumes based on infant BW and GA may decrease FI, which may be an issue of volume intolerance.

## Introduction

Newborns whose mothers intend to breastfeed may receive formula supplementation as early as their first feeding or later during their stay in the Well-Baby Nursery. Formula supplementation with the first feed is even more common in late preterm (LPT) infants (1, 2). In addition to maternal reasons, supplementation is employed for several reasons, such as perceived concerns of insufficient milk production, suboptimal intake, infant fussiness, excessive weight loss, hypoglycemia, hyperbilirubinemia, or even recommendations from healthcare providers (1, 3). However, there are no clear recommendations on the volume of supplementation a breastfed infant should receive on their first day of life. Even in exclusively formula-fed infants, although the American Academy of Pediatrics (AAP) states that “formula-fed babies will feed 2 oz every 3 to 4 h for the first 3 month,” there is not much clarity on the appropriate volume a newborn should receive within their first 24 h of life (24 HOL) (4).

Even if formula is used, the ideal feeding goals immediately after birth should be either to compensate for the deficit or to match the natural breast milk production in the early postpartum days. In reality, infants are often fed more than this physiological volume. These feeding volumes can even exceed the normal physiological stomach capacity of neonates based on the fetal and postnatal measurements of gastric volumes (5–9). Furthermore, during this early neonatal stage, neither the birth weight (BW) nor the gestational age (GA) is generally considered to decide the feeding volume of the infants. In addition, the emesis or fussiness of the baby is often perceived as feeding intolerance (FI), resulting in formula switching to a “sensitive” one. Whether these volumes in relation to infant BW and GA are associated with FI, including frequent formula switching, has not been well quantified. We hypothesized that infants who are either exclusively formula fed or receive hybrid feeding (breastfeeding with supplementation) are more likely to experience FI based on the feeding volumes in the first 24 HOL. In this study, we also explored feeding practices in relation to maternal socioeconomic and perinatal factors along with comorbidities in an inner-city hospital.

## Materials and methods

### Study design

This retrospective study was conducted in the mother–baby unit at Hurley Medical Center. The study population consisted of all the infants born at ≥35 weeks of GA between March 2018 and March 2021. Infants were excluded from the study if they were out born, had a length of stay less than 24 h, and had known congenital malformations or chromosomal abnormalities. The cases in our study were defined as infants with FI evidenced by chart documentation of emesis, abdominal distension, abdominal x-ray, and/or switching to a sensitive formula within the first 24 HOL. The institutional review board of Hurley Medical Center approved this study (1744664–1).

### Data collection

Data were collected from electronic medical records. The age of the mother, self-declared race, marital status, parity, insurance, mode of delivery [vaginal or cesarean section (CS)], use of general anesthetic at the time of delivery, use of illicit drugs, psychiatric illnesses, hypertension, pre-eclampsia, eclampsia, diabetes mellitus, and medication use during the current pregnancy was collected. The GA, BW, sex, timing of first feed, type of feedings (breastfeeding or formula), feeding volume, documentation of emesis, abdominal distension, x-ray imaging, switching to a sensitive formula, point of care (POC) glucose level and collection time, bilirubin levels, and weight loss at 24 HOL of the babies were recorded.

### Newborn feeding protocol

Feedings are documented according to the standardized protocol at our institution. Neonatal feeding is commenced as soon as possible after delivery as per a maternal feeding plan. Infants are fed within 60 min by either initiating nursing on the breast or giving formula milk if breastfeeding is contraindicated or mothers choose not to breastfeed. If breastfeeding is delayed due to maternal medical reasons, infants are offered formula initially, after getting maternal consent, and breastfeeding is resumed as soon as possible. The POC glucose level is checked at least 30 min after feeding in high-risk infants, as recommended in the AAP guidelines (10). It is also checked if the first feeding is delayed for any reason. Formula supplementation is offered if the POC level is <40 mg/dl post breastfeeding. The volume of formula supplementation or formula feeding at our institution is variable, mainly based on bedside nursing or maternal discretion. In the event of symptomatic hypoglycemia or asymptomatic hypoglycemia post feeding, IV glucose is started in accordance with AAP guidelines (10). In our institution, Similac Pro-Advance (20 kcal/oz) is the first choice of formula for all infants. If the bedside caregiver perceives FI, formula is switched to Similac Sensitive (20 kcal/oz) either on parental request or after consultation with the medical team.

### Categorization of the study population

**Figure 1:**
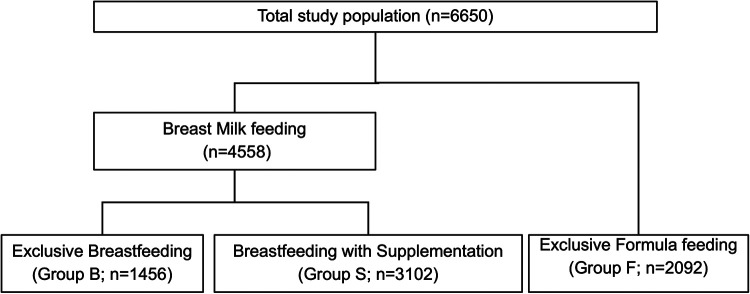
Flow diagram of study population. The flow chart depicts the mother and infant charts included in the final analysis based on data available on neonatal feeding types.

All infants born at ≥35 weeks of GA with documented feedings were included in the study and final analysis (Figure 1). Feeding types and volumes (if available) were collected from the charts of the infants. The study population was categorized into three groups based on the feeding documentation in the chart of the infant, namely, (a) exclusively breastfeeding (Group B), (b) exclusively formula feeding (Group F), or (c) hybrid feeding (breastfeeding with formula supplementation) (Group S), during the first 24 HOL. Neonates were also subcategorized into high-risk infants if they were LPT, small for gestational age (SGA), large for gestational age (LGA), or infant of diabetic mothers (IDM). Feeding characteristics were used to analyze the overall feeding profile and its association with neonatal FI and “breastfeeding at discharge.” *In our analysis, all the volumes of feed were calculated based on the neonate BW, and the feeding volumes were represented as ml/kg*.

### Statistical analysis

Baseline characteristics were compared based on the feeding documentation of the infants. Univariate analysis of continuous variables was performed using the *t*-test and ANOVA or the Kruskal–Wallis test based on data distribution, while categorical variables were evaluated using *χ*^2^ tests. Data were presented as either mean ± standard deviation (SD) or median [interquartile range (IQR)] for continuous variables and number (percentage) for categorical variables. We initially examined the maternal and neonatal characteristics among the three groups and subsequently between Groups B and S. We calculated the feeding volumes in milliliters in relation to BW in kilograms (ml/kg) for each formula feed during 24 HOL and correlated these with GA. Multiple logistic regression analysis correcting for confounding variables (maternal race; mode of delivery; use of anesthesia at delivery; maternal marital status; maternal insurance; parity; maternal disease: diabetes, hypertension, and psychiatric illness; neonatal prematurity; and gender) was performed to predict breastfeeding supplementation in the first 24 HOL and to analyze the associations of neonatal FI and breastfeeding at discharge (dependent variables) with feeding volumes. Based on our regression models, we performed post-estimation analyses to depict the predicted probability of our outcomes. A *p*-value of <05 was considered statistically significant. All statistical analysis was performed using StataCorp 2015 (Stata Statistical Software: Release 14, StataCorp LP).

## Results

### Study population and demographics

The medical records of 6,650 infants were reviewed. Overall, the mean GA at delivery was 38.8 ± 1.2 weeks, and BW was 3,217 ± 475 g. In total, 68.6% of neonates were delivered vaginally, 55.1% were Caucasian (CAU), and 51.1% were male. In addition, 9.0% were SGA and 5.2% were LGA. A total of 56,498 feeding data points were available, out of which 29,833 were of formula feeding. Overall, 1,765 (26.5%) infants had documentation of one or more types of FI, which included emesis (*n* = 958; 14.4%), formula change (*n* = 1,065; 21.4%), and/or abdominal distension and x-ray (54; 0.8%).

### Comparative analysis of maternal characteristics

Of the total study population, 1,456 infants (21.9%) exclusively breastfed (Group B), 3,102 (46.6%) were supplemented with breastfeeding (Group S), and 2,092 (31.5%) were exclusively formula-fed (Group F) (Figure 1). Overall, African American (AA) mothers had lower rates of breastfeeding (55.9% vs. 78.8%; *p* < 0.0001) and higher rates of hybrid feeding (79.3% vs. 61.5%; *p* < 0.0001). Table 1 presents other maternal profiles within these three groups. The multivariate logistic regression analysis of maternal perinatal characteristics associated with breastfeeding supplementation (Table 2) revealed that the odds of supplementation were higher among mothers who were AA, young, single, and primiparous and those with Medicaid insurance. Other significant factors include maternal diabetes, hypertension, psychiatric illness, delivery by CS, and use of anesthesia.

**Table 1:** Baseline variable in mothers and infants based on type of feeding in the first 24 h of life.

Variables	Group B^a^ (*n = *1,456)	Group S^a^ (*n = *3,102)	Group F^a^ (*n = *2,092)	*p*-value
Maternal age, mean (SD), years	28.8 (5.2)	27.8 (5.6)	26.3 (5.3)	<0.001
African American race, *n* (%)	346 (23.8)	1,326 (42.7)	1,317 (63)	<0.001
Primiparous, *n* (%)	77 (5.3)	235 (7.6)	86 (4.1)	<0.001
Single/divorced, *n* (%)	671 (46.1)	1,925 (62.1)	1,802 (86.1)	<0.001
Medicaid insurance, *n* (%)	678 (46.6)	1,818 (58.6)	1,816 (86.8)	<0.001
Diabetes, *n* (%)	109 (7.5)	369 (11.9)	221 (10.6)	<0.001
Hypertension, *n* (%)	149 (10.2)	464 (15.0)	329 (15.7)	<0.001
Anemia, *n* (%)	215 (14.8)	597 (19.2)	545 (26.1)	<0.001
Psychiatric illness, *n* (%)	404 (27.7)	1,045 (33.7)	775 (37.0)	<0.001
History of illicit drug use, *n* (%)	148 (10.4)	416 (13.6)	555 (26.9)	<0.001
History of smoking, *n* (%)	1,098 (76.0)	2,319 (75.2)	1,528 (73.1)	0.112
Delivery by C-section, *n* (%)	361 (24.8)	1,091 (35.2)	634 (30.3)	<0.001
Epidural anesthesia, *n* (%)	1,058 (73.3)	2,447 (79.8)	1,538 (74.7)	<0.001
General anesthesia, *n* (%)	24 (1.7)	132 (4.3)	91 (4.4)	<0.001
Gestational age, mean (SD), week	39.1 (0.9)	38.7 (1.2)	38.6 (1.2)	<0.001
Birth weight, mean (SD), g	3,368 (411)	3,209 (499)	3,124 (453)	<0.001
Birth weight, mean (SD), *z*-score	0.01 (0.8)	−0.1 (0.9)	−0.3 (0.8)	<0.001
Male sex, *n* (%)	706 (48.5)	1,496 (51.8)	1,007 (51.9)	0.081
Small for gestational age, *n* (%)	74 (5.1)	299 (9.6)	227 (10.9)	<0.001
Large for gestational age, *n* (%)	75 (5.2)	209 (6.7)	65 (3.1)	<0.001
Time to 1st feed (minutes), mean (SD)	49.0 ± 23.7	56.9 ± 22.8	56.0 ± 24.9	<0.001

^a^ Group B, exclusive breastfeeding; Group S, breastfeeding with supplementation; Group F, exclusive formula feeding.

**Table 2:** Multivariate logistic regression analysis: perinatal characteristics predicting breastfeeding supplementation.

Variable	Group B*	Group S*	OR	95% CI	*p*-value
	*n* = 1,456 (21.9%)	*n* = 3,102 (46.6%)			
Race (AA)^a^	19.6%	37.5%	2.180	1.865–2.546	**<0.001**
Maternal age 15–19 years^b^	1.9%	4.9%	2.274	1.473–3.509	**<0.001**
Maternal age 20–25 years^b^	19.6%	27.1%	1.299	1.101–1.532	**0.002**
Single/divorced^c^	46.1%	62.0%	1.309	1.113–1.540	**0.001**
Medicaid insurance^d^	46.6%	58.6%	1.242	1.062–1.454	**0.007**
Primiparous^e^	66.7%	70.3%	1.201	1.028–1.409	**0.021**
Diabetes	6.8%	9.8%	1.350	1.148–1.587	**<0.001**
Hypertension	10.2%	14.9%	1.336	1.079–1.653	**0.008**
Psychiatric illness	27.7%	33.7%	1.245	1.069–1.449	**0.005**
History of illicit drug use	10.3%	13.6%	1.056	0.846–1.317	0.63
Delivery by C-section^f^	24.8%	35.2%	1.400	1.184–1.654	**<0.001**
Epidural anesthesia^g^	73.3%	79.8%	1.601	1.342–1.910	**<0.001**
General anesthesia^g^	1.7%	4.3%	2.681	1.606–4.474	**<0.001**
Late preterm (35–37 weeks)^h^	1.0%	8.1%	9.933	5.626–17.53	**<0.001**
SGA^i^	5.1%	9.6%	1.751	1.322–2.319	**<0.001**
LGA^i^	5.2%	6.7%	1.496	1.115–2.009	**0.007**

Group B, exclusive breastfeeding; Group S, breastfeeding with supplementation; AGA, appropriate for gestational age.

Variables included in the regression include volume of supplementation; maternal race; mode of delivery; use of anesthesia at delivery; maternal marital status; maternal insurance; parity; maternal disease: diabetes, hypertension, and psychiatric illness; prematurity; and birth weight.

Bold indicates *p* value <0.05.

^a^ Maternal race (white as referent).

^b^ Maternal age (>25 years as referent).

^c^ Marital status (married as referent).

^d^ Maternal insurance (private insurance as referent).

^e^ Parity (multiparity as referent).

^f^ Mode of delivery (vaginal as referent).

^g^ Anesthesia (none as referent).

^h^ Prematurity (GA > 37 weeks as referent).

^i^ Neonatal weight (AGA as referent).

### Feeding volume and FI

**Figure 2:**
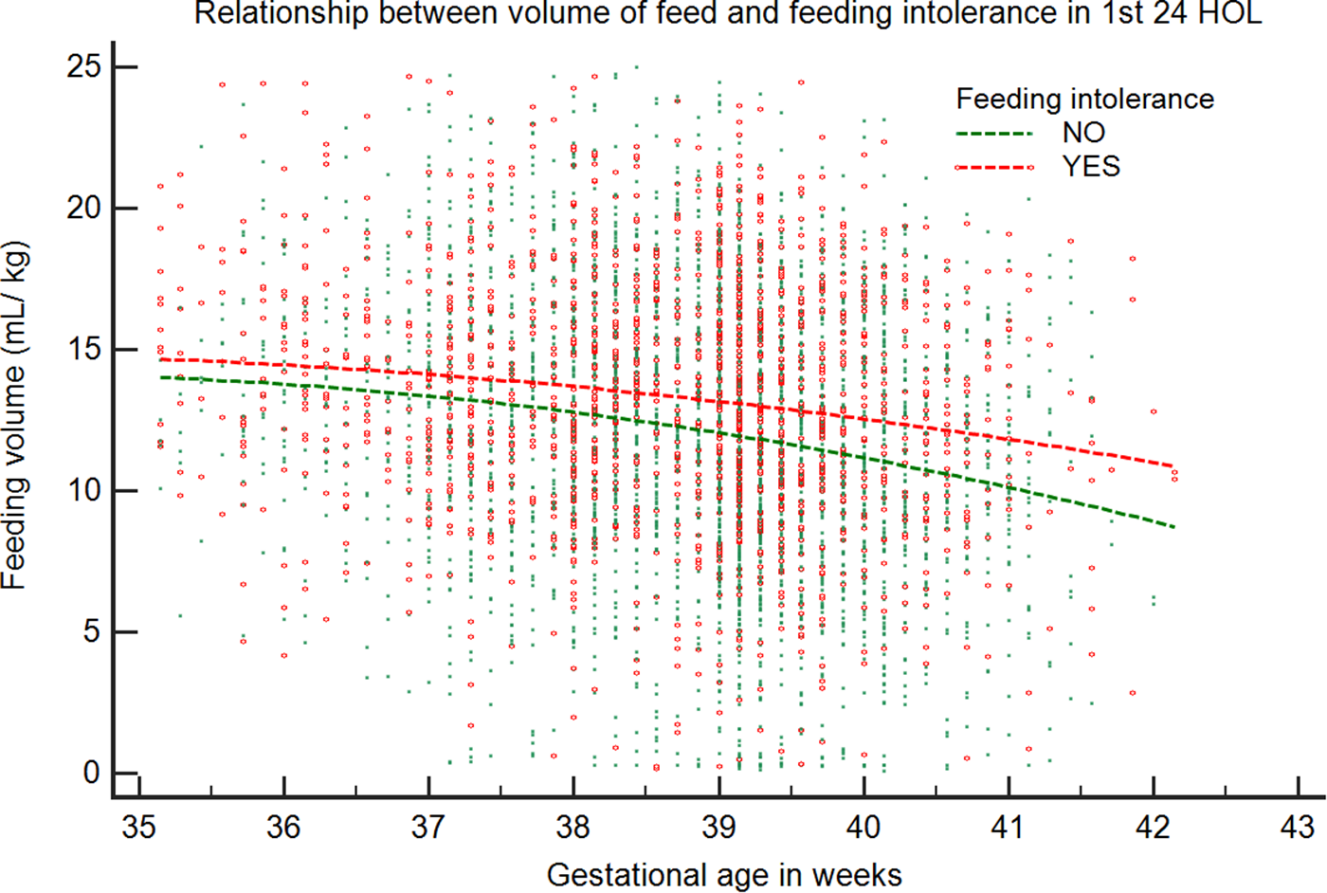
Relationship between maximum volume of feed in relation to gestational age in the first 24 HOL. The volume of feed (ml/kg) is plotted against GA for infants with and without feeding intolerance. Data representative of 5,104 infants (infants without feeding problems: 3,459; infants with feeding problems: 1,645).

**Figure 3:**
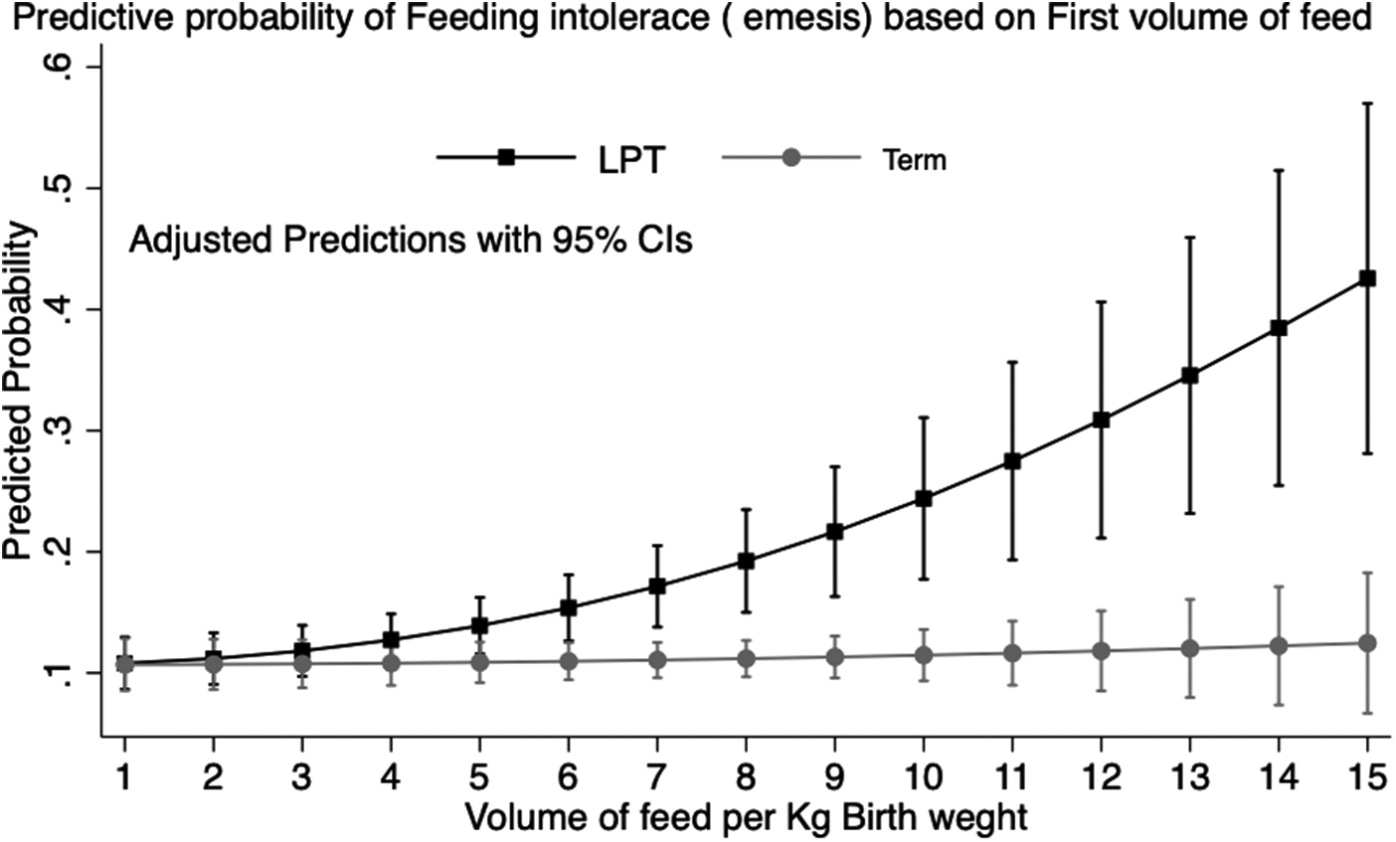
Prediction of feeding intolerance (emesis) by volume of first feed: predictive margins with 95% CI plotted by prematurity in healthy newborn babies in the well-baby nursery. Data representative of 2,610 infants (LPT: 286; term: 2,324).

Overall, the maximum volume of formula intake (ml/kg) per feed was inversely correlated with GA and was higher in infants with FI (*β* = −1.39, *p* < 0.001) compared with those without FI (*β* = −1.28, *p* < 0.001) (Figure 2). We looked at infants who received their first feeding with formula milk [*n* =** **2,610 (39.2%)]. The values were significantly higher in LPT infants than in term infants (54.7% vs. 38.2%; *p* < 0.001). The median intake of the first feed was 7.4 ml/kg (IQR: 7.2–7.5). Among LPT infants, the first formula feeding was associated with a higher incidence rate of emesis compared with that of term infants (20.5% vs. 12%; *p* = 0.01), with a higher median intake in ml/kg in the emesis group (8.1 vs. 6.9; *p* = 0.01). After adjusting for confounding variables, the first feeding volume >8 ml/kg was associated with higher odds of emesis [odds ratio (OR) = 2.5, 95% confidence interval (CI): 1.4–4.6; *p* = 0.002]. The post-estimation predictive probability of emesis in relation to the volume of the first feed showed a positive correlation between volume and emesis (Figure 3).

**Figure 4:**
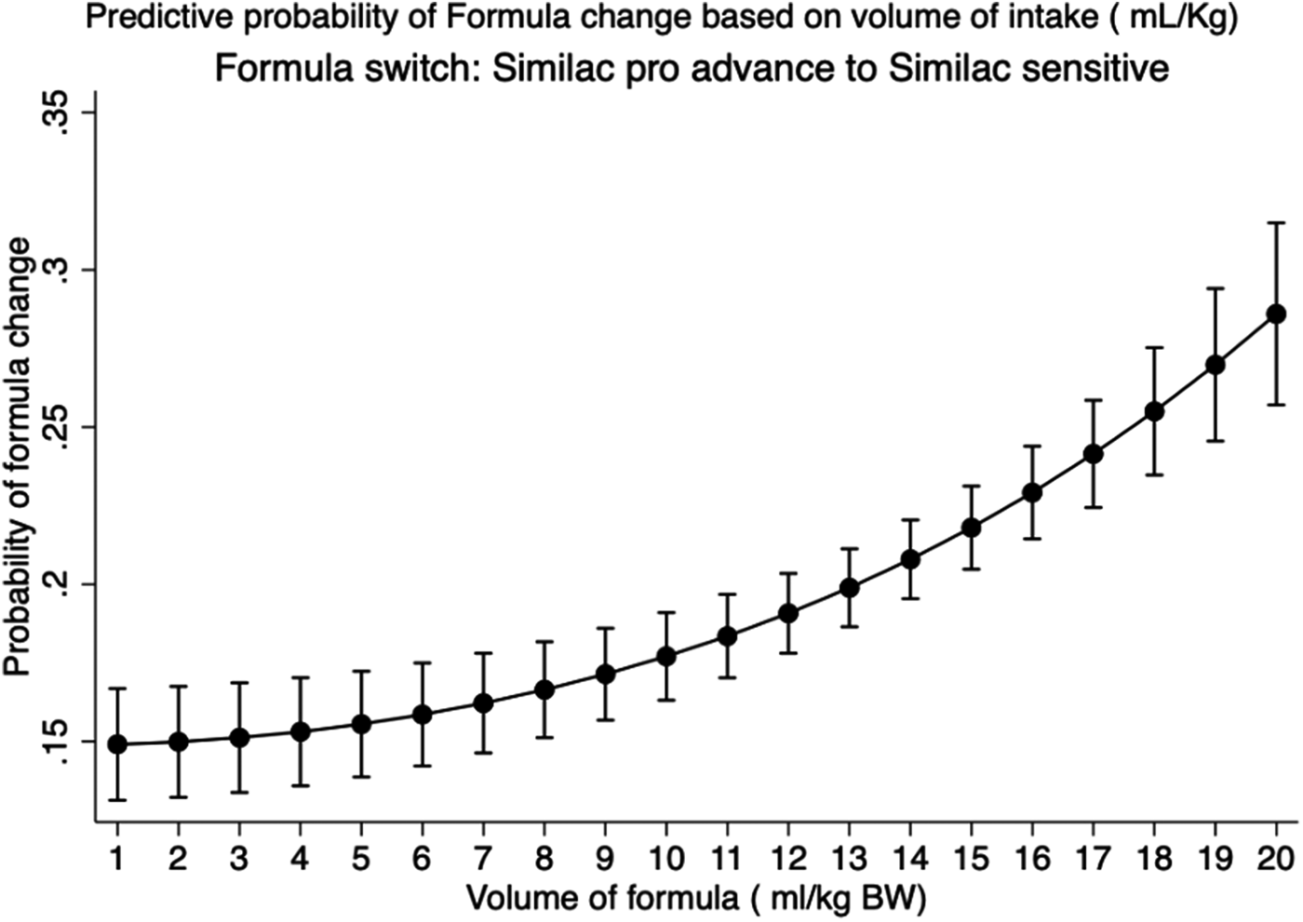
Prediction of switching of regular formula to a sensitive formula based on volume of intake in exclusive formula-fed infants: predictive margins with 95% CI plotted in healthy newborn babies in the Well-Baby Nursery who were exclusively formula-fed. Data representative of 2,092 infants.

The median intake of the maximum volume of formula per feed for the entire Group F cohort was 14.3 ml/kg (IQR: 11.7–17.0). There were more AA infants in Group F. While the median BW of the AA group was slightly lower than that of the CAU group [3.1 kg (2.8–3.4) vs. 3.2 kg (2.9–3.5); *p* < 0.01], more AA infants received volumes higher than the median compared with CAU infants (59.3% vs. 40.1%; *p* < 0.001). Emesis was documented in 10.4% of Group F compared with 2.9% of Group B (*p* < 0.001). In total, 27.0% of infants in Group F were switched to sensitive formula (Similac Sensitive). Based on receiver operator curve analysis (AUC 0.6; *p* < 0.001), infants receiving >10.5 ml/kg had higher odds of formula switch [OR: 2.2 (95% CI: 1.8–2.6)]. Figure 4 depicts the post-estimation predictive probability of “*switching to a sensitive formula*.” Changes in BW related to formula feeding were also analyzed. Compared with Group B, the maximum weight loss (%) in Group F was lower (4.3 ± 2.1 vs. 2.5 ± 2.0; *p* < 0.001). In addition, a higher percentage of infants gained weight in Group F over the first 24 h (11.3% vs. 4.4%; *p* < 0.001).

**Figure 5:**
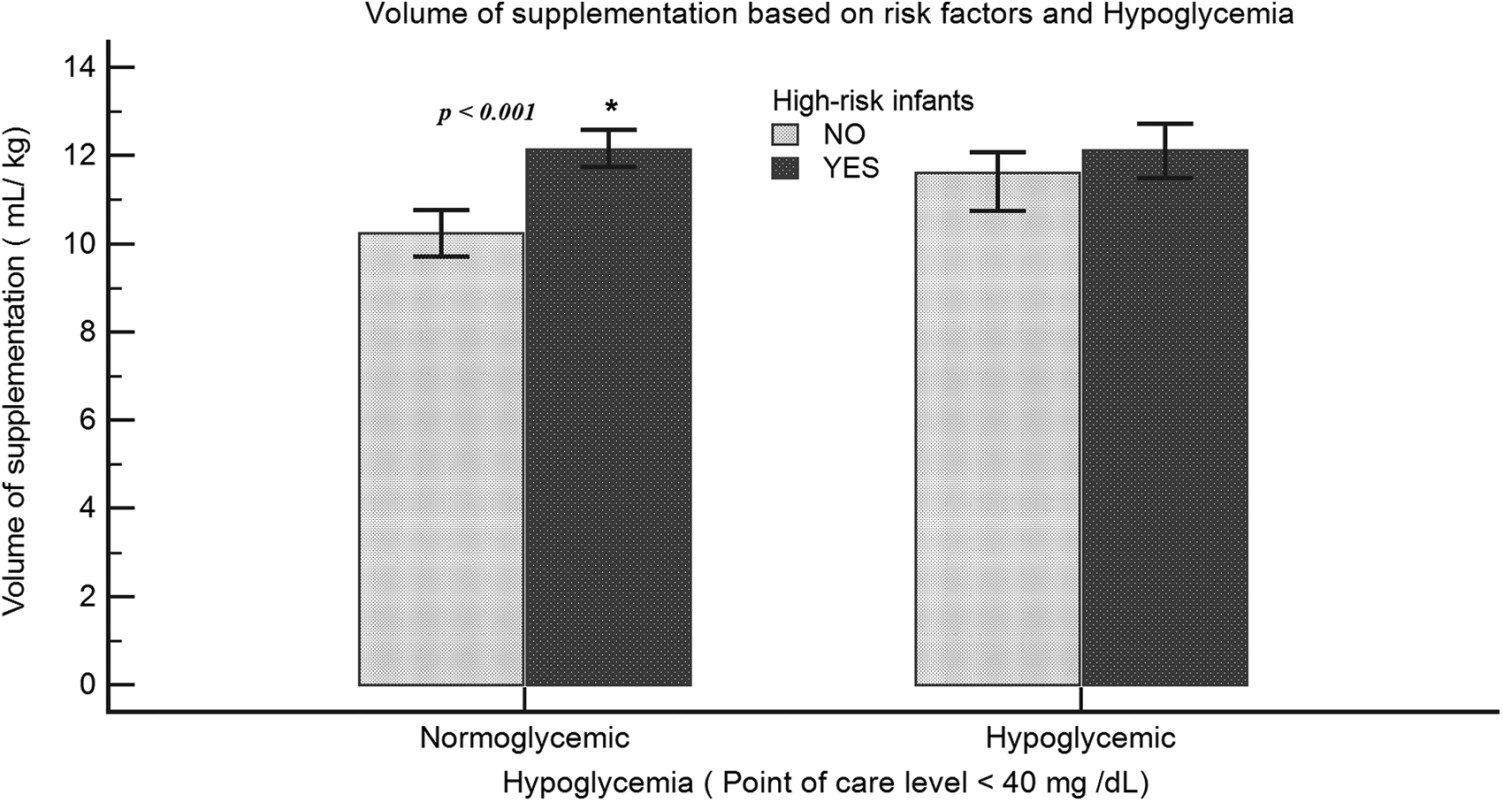
Volume of supplementation among high-risk infants: volume of intake (ml/kg) was compared among neonates with or without risk for hypoglycemia. High-risk infants include LPT, SGA, LGA, or IDM. Data representative of 1,608 infants (normoglycemia: no risk factors, *n* = 539; risk factors, *n* = 502; hypoglycemia: no risk factors, *n* = 154; risk factors, *n* = 413).

The supplementation characteristics in breastfed infants who were supplemented (Group S) were as follows: Although most mothers chose to breastfeed their infants, a large proportion of infants (68%) were supplemented. The incidence rate of FI was higher in Group S compared with Group B (20.4% vs. 2.9%; *p* < 0.001). Among Group S infants, FI was associated with a higher median volume intake in milliliter/kilogram (12.4 vs. 10.6; *p* < 0.001). After adjusting for confounding variables, there was an incremental increase in the odds of FI with formula volume per feed of 5–10 ml/kg (OR = 2.8, 95% CI: 1.9–4.3), 10–15 ml/kg (OR = 4.7, 95% CI: 3.1–6.8), and >15 ml/kg (OR = 5.2, 95% CI: 3.4–7.9). The odds of FI were also higher among AA and LPT infants (Table 3). The documented reason for supplementation was only present in 24% of the charts. In Group S, only 18.4% had documented hypoglycemia (POC < 40 mg/dl) prior to supplementation, 34.5% had POC levels within the normal range, and 47.1% had no POC levels checked. Among the high-risk infants (SGA, LGA, LPT, and IDM), the volume intake was significantly higher even in the absence of hypoglycemia (10.2 vs. 12.1 ml/kg; *p* < 0.001) (Figure 5). We also analyzed the available POC levels (*n* = 1,290) among infants with no documented risk factors. Although the median POC (mg/dl) levels were lower in the breastfeeding groups [52 (44–62) vs. 59 (50–69); *p* < 0.001] compared with Group F, the percentage of infants requiring intravenous dextrose was not statistically significant (6.6% vs. 3.8%; *p* = 0.055).

**Table 3:** Multivariate logistic regression analysis: predicting feeding volume intolerance with breastfeeding supplementation.

Variable	OR	95% CI	*p*-value
Volume of formula intake (ml/kg)^a^			
5–10 ml/kg	2.887	1.922–4.337	**<0.001**
10–15 ml/kg	4.664	3.114–6.896	**<0.001**
>15 ml/kg	5.207	3.417–7.934	**<0.001**
Race (CAU)^b^	0.720	0.580–0.894	**0.003**
Maternal age 15–19 years^c^	0.911	0.589–1.408	0.677
Maternal age 20–25 years^c^	1.025	0.829–1.267	0.819
Single/divorced^d^	0.993	0.772–1.277	0.958
Medicaid insurance^e^	1.129	0.896–1.422	0.302
Primiparous^f^	0.914	0.724–1.154	0.452
Diabetes	0.904	0.647–1.265	0.559
Hypertension	0.918	0.401–2.100	0.840
Psychiatric illness	1.122	0.910–1.384	0.279
History of illicit drug use	1.279	0.973–1.682	0.077
Delivery by C-section^g^	0.986	0.788–1.235	0.909
General anesthesia^h^	1.645	1.015–2.666	0.043
Late preterm (35–37 weeks)^i^	1.815	1.292–2.550	**0.001**
SGA^j^	1.107	0.802–1.528	0.535
LGA^j^	1.299	0.872–1.933	1.197

The variables included in the regression are volume of supplementation; maternal race; mode of delivery; use of anesthesia at delivery; maternal marital status; maternal insurance; parity; maternal disease: diabetes, hypertension, and psychiatric illness; prematurity; and birth weight.

Bold indicates *p* value <0.05.

^a^ Volume of formula intake (<5 ml/kg as referent).

^b^ Maternal race (White as referent).

^c^ Maternal age (>25 years as referent).

^d^ Marital status (married as referent).

^e^ Maternal insurance (private insurance as referent).

^f^ Parity (multiparity as referent).

^g^ Mode of delivery (vaginal as referent).

^h^ Anesthesia (none as referent).

^i^ Prematurity (GA > 37 weeks as referent).

^j^ Neonatal weight (AGA as referent).

## Discussion

In this study, we analyzed the feeding volumes of newborns within the first 24 HOL. This research is one of the first studies to examine the relationship between ml/kg milk intake at each feeding and the occurrence of FI. Strikingly, we noted an inverse relationship between higher volumes of ml/kg feeding and GA, and this relationship was stronger in infants with documented FI. Our data also showed a positive correlation between feed volume and formula switching. This association was more pronounced among LPT infants. Although our findings were obtained from one center, they suggested that volumes >8 ml/kg/feed in the first 24 h of life can potentially cause FI.

One of the many reasons caregivers choose to supplement is the perceived inadequate intake, aiming to ensure that the infant is satisfied and gets “enough milk” (3). This perception often leads to higher feeding volumes, ignoring infant BW and GA, which may not be tolerated well immediately after birth. Our correlation of feeding volumes/kilogram BW and GA clearly demonstrates this pattern. Our data also show that the odds of FI increased by 2.8-, 4.6-, and 5.2-fold with 5–10, 10–15, and >15 ml/kg of supplementations, respectively. Our findings can probably be explained by the stomach capacity in the early postnatal phase as extrapolated from the measured fetal stomach sizes. Based on fetal ultrasound measurements (*n* = 152), the data of Goldstein et al. (11) suggest a gastric volume of 12 ml at 37–39-week GA. Widström et al. (12) measured the volume of aspirated contents of the stomach, which was 10 ml, “immediately after birth” in term infants (*n* = 25). Other studies using intragastric pressures and autopsy findings suggested slightly higher volumes, varying between 15 and 30 ml (8). After compiling all these findings, the stomach size at birth was estimated at approximately 20 ml (8). The sensitivity of newborns to these higher volumes is probably a reflection of the relatively small stomach capacity at birth. Based on the stomach size estimates of 20 ml, volumes of >7–10 ml/kg are unlikely to be tolerated immediately after birth, which is confirmed by our data showing FI increases in a dose-dependent manner in infants fed 5–10, 10–15, and 15–30 ml/kg/feed. Volume sensitivity was also observed among exclusively formula-fed infants. We found that the probability of needing a formula change due to reported FI increased as much as 40% when the infant was fed large volumes (>10 ml/kg) of formula. However, recent studies had also reported adverse outcomes of overfeeding, specifically 30 ml or more per feed, in term infants on the first day (5, 8). Our data showed that volume considerations in relation to BW may be more sensitive in predicting FI during the first days of life. One of the limitations of our study is the accuracy of feed volumes, which was often reported by mothers at the bedside, introducing the potential for errors.

LPT infants are known to be at a higher risk of receiving formula milk after birth, which may be secondary to maternal reasons such as cesarean section and use of anesthesia, hypertension, pre-eclampsia, and lower blood sugars. Our data show that LPT infants are even more sensitive to volume intake. First feeding volumes of >8 ml/kg increase the odds of emesis by 2.5-fold. This higher sensitivity may be because gastric volumes seem to increase rapidly from 35–36 (∼7 ml) to 39 (∼12 ml) white globe appearance based on the measurements of recent fetal ultrasounds (6, 7). These findings suggest that extra caution is required when initial feedings are offered to these smaller and less mature infants. We acknowledge that emesis/spit-ups are not always accurately or consistently documented, and there may be underreporting in the chart of the infant.

Although AAP does not recommend supplementation without medical indication (13), we found that among the breastfeeding supplementation group, only 18.4% have documented hypoglycemia prior to supplementation. In contrast, about a third of the infants are euglycemic, and almost half are supplemented without POC checks. Furthermore, supplementation volume is significantly higher among high-risk infants even without hypoglycemia.

Feeding infants more than natural breast milk production during the early postpartum phase might also affect physiological weight loss typically observed after birth. Although the average weight loss was in the normal range (average of 4.3%) in the breastfeeding groups, 11.3% gained weight among exclusively formula-fed infants, which may not be physiologically appropriate. While our center had guidelines for supplementation based on hypoglycemia (based on AAP guidelines), we did not have specific guidelines for volumes of supplementation with every feed. This practice contributed to our results of potentially excessive feeding but was likely similar to numerous practices across the country, as guidelines did not exist.

We found that AA mothers had higher rates and volumes (ml/kg) of formula feedings when compared with CAU mothers. Among breastfeeding mothers, the rates of supplementation were both significantly higher and highly variable. Based on the recent National Immunization Survey (NIS) reports from the Centers for Disease Control (CDC), there was a significant racial and ethnic disparity in breastfeeding rates (14). Our data were consistent with these reports. However, we observed a significantly higher breastfed supplementation rate of 68%, contrasting with the NIS-reported rates of 19.2%. This discrepancy might be due to the nature of data collection of this phone survey conducted 19–35 months post-delivery, with relatively lower response rates (21%–33%) from parents, which might lend it to recall bias. Furthermore, our finding is consistent with the recently published data on formula supplementation from 126 New York hospitals with over 150,000 breastfed infants. This study found 50.6%–57.0% supplementation rates in higher-level hospitals, similar to those in our institution (2). In addition, consistent with these previous reports, we found that supplementation was significantly higher with AA race, Medicaid insurance, single mother, cesarean delivery, and maternal comorbidities such as diabetes, hypertension, and psychiatric illness. The reasons for racial differences in the increase in supplementation in AA mothers are unclear but are likely multi-factorial.

Early and exclusive breastfeeding has been recommended as the preferred and optimum source of infant nutrition (15–17). Early formula feeding has been negatively associated with subsequent breastfeeding rates (1, 2, 4, 13, 18–20), besides several other adverse outcomes implicated with formula use in infants (21–29). Future studies will be needed to determine if volumes higher than the natural breast milk production during the early postpartum stage can also potentially discourage mothers from continuing breastfeeding both in the hospital and after discharge and hence may be a factor impacting breastfeeding outcomes. Although this was a retrospective single-center study, according to CDC's Maternity Practices in Infant Nutrition and Care (mPINC) survey data on the statement of “*few breastfeeding newborns receive infant formula*,” only 38% (nationally) and 27% (Michigan) of hospitals had an ideal response (30). On the statement of “*when breastfeeding mothers request infant formula, staff counsel them about possible consequence*,” 61% (nationally) and 30% (Michigan) of hospitals had an ideal response (30). Our data highlighted the fact that still much more needs to be done to achieve the desired goals of optimal neonatal breastfeeding.

## Conclusion

This research is one of the first studies to demonstrate a birth weight–dependent relationship between neonatal feeding and feeding intolerance. Although this is a single-center study, our large data set indicates that feeding volumes per kilogram BW may impact important early neonatal feeding outcomes. We clearly demonstrate that supplementation volumes >5 ml/kg/feed during the first 24 HOL in neonates are associated with non-linear increases in volume intolerance, and importantly, volumes >10 ml/kg increase the probability of intolerance by as much as 40% in exclusively formula-fed babies. Despite the recommendations from both AAP and Academy of Breastfeeding Medicine (ABP), breastfeeding supplementation continues to be significantly higher (31–34). Our data show a much higher rate of supplementation and significantly higher volumes of intake, which increases the risk of FI. Furthermore, we also found that the reasoning for supplementation was not properly documented. We believe that a good proportion of the FI in the first 24 HOL may be related to non-physiologic feeding practices, leading to higher volume intakes, and hence should be called “volume intolerance.” We also believe that standardizing feeding volumes in the newborn nursery (NBN) can also potentially decrease supplementation rates, as one of the reasons for supplementation is the perception that “*my baby is not getting enough*.” Our data lend credence to the fact that early neonatal stomach capacity is small and hence should be considered while feeding our newborns in the NBN.

## Data Availability

The raw data supporting the conclusions of this article will be made available by the authors, without undue reservation.
